# How Clonal Is Clonal? Genome Plasticity across Multicellular Segments of a “*Candidatus* Marithrix sp.” Filament from Sulfidic, Briny Seafloor Sediments in the Gulf of Mexico

**DOI:** 10.3389/fmicb.2016.01173

**Published:** 2016-08-03

**Authors:** Verena Salman-Carvalho, Eduard Fadeev, Samantha B. Joye, Andreas Teske

**Affiliations:** ^1^HGF MPG Joint Research Group for Deep-Sea Ecology and Technology, Max Planck Institute for Marine MicrobiologyBremen, Germany; ^2^Department of Marine Sciences, University of GeorgiaAthens, GA, USA; ^3^Department of Marine Sciences, University of North Carolina at Chapel HillChapel Hill, NC, USA

**Keywords:** “*Candidatus* Marithrix”, filamentous sulfur bacteria, polyploidy, single nucleotide polymorphism, microevolution

## Abstract

“*Candidatus* Marithrix” is a recently described lineage within the group of large sulfur bacteria (*Beggiatoaceae, Gammaproteobacteria*). This genus of bacteria comprises vacuolated, attached-living filaments that inhabit the sediment surface around vent and seep sites in the marine environment. A single filament is ca. 100 μm in diameter, several millimeters long, and consists of hundreds of clonal cells, which are considered highly polyploid. Based on these characteristics, “*Candidatus* Marithrix” was used as a model organism for the assessment of genomic plasticity along segments of a single filament using next generation sequencing to possibly identify hotspots of microevolution. Using six consecutive segments of a single filament sampled from a mud volcano in the Gulf of Mexico, we recovered ca. 90% of the “*Candidatus* Marithrix” genome in each segment. There was a high level of genome conservation along the filament with average nucleotide identities between 99.98 and 100%. Different approaches to assemble all reads into a complete consensus genome could not fill the gaps. Each of the six segment datasets encoded merely a few hundred unique nucleotides and 5 or less unique genes—the residual content was redundant in all datasets. Besides the overall high genomic identity, we identified a similar number of single nucleotide polymorphisms (SNPs) between the clonal segments, which are comparable to numbers reported for other clonal organisms. An increase of SNPs with greater distance of filament segments was not observed. The polyploidy of the cells was apparent when analyzing the heterogeneity of reads within a segment. Here, a strong increase in single nucleotide variants, or “intrasegmental sequence heterogeneity” (ISH) events, was observed. These sites may represent hotspots for genome plasticity, and possibly microevolution, since two thirds of these variants were not co-localized across the genome copies of the multicellular filament.

## Introduction

Filamentous large sulfide-oxidizing bacteria of the *Gammaproteobacteria* occur frequently in sulfide-rich estuarine and coastal marine sediments, and in the deep sea at vent and seep sites (Jørgensen, 1977; Jannasch and Wirsen, 1981; Williams and Reimers, 1983; Jannasch et al., 1989; Nelson et al., 1989; Larkin and Henk, 1996; McHatton et al., 1996; Kojima and Fukui, 2003; Heijs et al., 2005; Jørgensen et al., 2010; Grünke et al., 2011, 2012; McKay et al., 2012; MacGregor et al., 2013a). These microorganisms form mats on top of and within the first centimeters of sulfidic sediments. They utilize sulfide that either diffuses upwards from the underlying sulfate reduction zone, or that is transported in the advective flow of reduced discharging fluids. Elemental sulfur is stored internally as globules, and serves as energy reserve. Free-living, gliding filamentous representatives of this group are commonly observed in deep ocean cold and hot seep settings, and previous investigations on their ecology and genetic potential have revealed some insights into the specific adaptations to sulfide, oxygen, nitrate, and temperature gradients (Kojima and Fukui, 2003; Heijs et al., 2005; Grünke et al., 2011, 2012; McKay et al., 2012; MacGregor et al., 2013a).

DNA staining of large sulfide-oxidizers such as *Thiomargarita* (Schulz, 2006), *Beggiatoa* (Hinck et al., 2011), and *Achromatium* (Salman et al., 2015) reveals numerous fluorescent spots of various sizes. In the only other group of giant bacteria with similarly large cell biomass—the surgeon fish-symbionts affiliated with the genus *Epulopiscium* in the *Firmicutes—*a comparable degree of massive DNA accumulation was observed (Angert, 2006, 2012). Here, extreme polyploidy was tested and revealed up to 450,000 genome copies per cell, based on the counting of single-copy genes. The genome copy numbers were correlated linearly with cell size (Bresler and Fishelson, 2003; Mendell et al., 2008). Mendell et al. (2008) analyzed the conservation of three housekeeping genes within an individual *Epulopiscium* cell and observed an extremely high level of conservation, while the amounts of single nucleotide substitutions were within the error range of the used Taq DNA polymerase. For the large sulfide-oxidizing bacteria of the *Gammaproteobacteria*, genome copy numbers per cell have not yet been determined, but extreme polyploidy is assumed (Lane and Martin, 2010). Explanations for the excessive polyploidy in giant bacteria remain speculative, but considering the implications of giant cell size and diffusion limitation (Schulz and Jørgensen, 2001), an even distribution of genome copies across the giant cell body would allow functional compartmentalization and independent response to stimuli at any cell region (Mendell et al., 2008). For example, each *Epulopiscium* cell has one genome per 1.9 μm^3^ of cytoplasm, which is even ca. three times less than a “regular sized” *Bacillus subtilis* cell with one chromosome per 0.7 μm^3^ (Mendell et al., 2008).

Gene duplication is believed to lead to a relaxation of selection on one gene copy, which allows mutations that could lead to diversification and adaptation (Wendel, 2000). A consequence of this relaxed selection could be a higher probability of generating pseudogenes in polyploid organisms instead of the development of functionally diverse alleles (Walsh, 1995). A high copy number of genes could therefore increase the mutation rate of a particular gene and allow the accumulation of null mutations, thereby not necessarily increasing fitness of the organism (Otto, 2007). On the other hand, and as demonstrated with *Epulopiscium* (Mendell et al., 2008), there is also firm evidence that polyploidy can support a high rate of homologous recombination, leading to an extremely efficient conservation among the genomes. Experiments with polyploid tobacco plastid genomes revealed that polyploidy allows efficient DNA repair and balancing of genome copies (Khakhlova and Brock, 2006). This process is mediated by inter-locus recombination events (Friedberg, 2003), using the genomic copies as template for genetic lesions. Also in *Archaea*, some organisms show a rapid equalization of genomes in the absence of selection, indicating that gene conversion may be a result of polyploidy, and could represent an evolutionary advantage (Soppa, 2011). In young polyploid organisms, the alleles usually retain expression, whereas in older polyploids excess alleles are usually lost (Wendel, 2000; and references therein).

In this study, the conservation extent of the genomes and genome copies within a multicellular filament of “*Candidatus* Marithrix” was investigated. The working hypothesis specified that the cells within a filament are not only polyploid, but also clonal to each other, i.e., their entire genetic content is considered identical, because the cells are directly descended from each other and represent only a few coexisting, contemporaneous generations. “*Candidatus* Marithrix” organisms occur at seep sites, and were initially described as *Beggiatoa*-like mats (Kalanetra et al., 2004; Heijs et al., 2005; Kalanetra and Nelson, 2010; Grünke et al., 2011, 2012). The cells are ca. 100 μm in diameter, and form filaments that are up to several centimeters in length. The filaments are non-motile and have the ability to attach to surfaces (Kalanetra et al., 2004; Kalanetra and Nelson, 2010), such as rocks (Jacq et al., 1989; Kalanetra et al., 2004), benthic animals such as tube worms (Kalanetra and Nelson, 2010), and shells of benthic crustaceans and gastropods (Salman et al., 2013), where they can serve as a food source to grazing benthic gastropods (Stein, 1984). Here, we describe a “*Candidatus* Marithrix” population encountered in the vicinity of an active mud volcano at Green Canyon (GC246) in the Gulf of Mexico. We present the first genomic data for this interesting group of organisms, and besides providing insights into their potential ecophysiology, this sequencing approach allows for a case study to investigate the genome plasticity along segments of a single “clonal” filament as well as within a highly polyploid organism.

## Materials and methods

### Sampling

**Figure 1:**
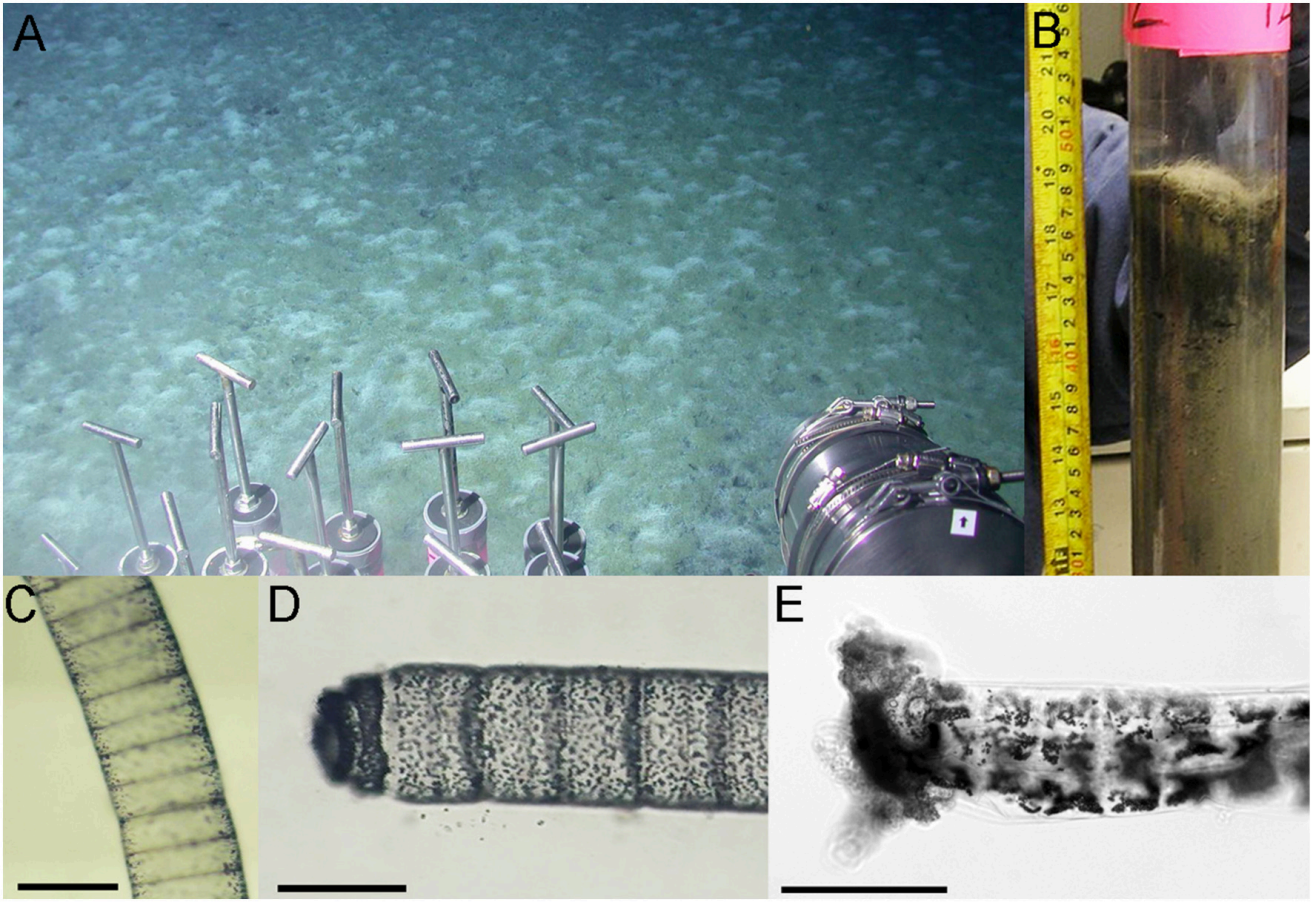
**Images of the microbial mats and “***Candidatus*** Marithrix” organisms. (A)** Image of the external camera of Alvin showing a field view of the microbial mats at GC246. **(B)** Original sediment core from the Marithrix microbial mat at GC246. Filaments are visible as hairy mat on top and within the first centimeter of the sediment. **(C)** Microscopic image of a filament showing small sulfur granules at the periphery of the disc-shaped cells, while the interior appears empty as they supposedly contain the large aqueous vacuole as described in other “*Candidatus* Marithrix” and related large sulfur bacteria. **(D)** Some of the freshly collected specimen showed an unusual filament end—not forming a typical semi-circle, but a differentially segmented cap; possibly a holdfast structure. **(E)** A freeze-thawed filament, which has lost most of its integrity, also showed an untypical ending on one side of the filament, possibly the holdfast structure with some sediment debris attached to it. Scale bars = 100 μm.

During cruise AT18-02 (Nov 2010) onboard the *R/V* Atlantis in the Gulf of Mexico, push cores (4562-9 to 4562-12) were taken with the submersible Alvin at a site where fluffy bacterial mats covered the seafloor sediment in a tuft-like pattern (Figure 1A). In the vicinity, small mud volcanoes and brine flows surrounded a brine pool (Green Canyon 246; depth 833 m; 27°42.089 N; 90°38.876 W). Individual filaments were retrieved from core 4562-12 (Figure 1B) with forceps, washed in *in situ* water, dragged through soft sterile agar, and washed again. Using a shipboard microscope, the diameter of the filaments was determined as ca. 110 μm (Figure 1C). Individually cleaned filaments were placed into cryovials containing 1 mL sterile DNAse-free water, frozen at −80°C, and transported back to the home lab. The cryovial was thawed on ice, and the intact filament was flushed carefully into a petri dish containing UV-treated MilliQ water. With the help of a stereomicroscope and sterile needles, the filament was cut into six segments, each containing ca. 30 cells. Individually, each segment was placed shortly into sterile MilliQ water, and was then added to the buffer for whole genome amplification.

### Whole genome amplification

The whole genome of each filament segment was amplified using the illustra GenomiPhi V2 DNA Amplification kit (GE Healthcare Life Sciences, Pittsburgh, PA) for multiple displacement amplification (MDA; Spits et al., 2006). Each filament segment was placed into 2.5 μL drops of sample buffer in a petri dish, manually disrupted with sterile metal needles, and pipetted into sterile 0.2 mL microcentrifuge tubes. Each buffer-cell-mix was boiled at 95°C for 3 min, amended by 0.2 μL reaction buffer and 0.5 μL V2 enzyme mix, and incubated at 30°C for 2 h before the reaction was terminated at 65°C for 10 min. The success of the six amplifications was visualized on a 0.8% agarose gel, and the purity of the amplicons were tested by 16S rRNA gene sequencing and phylogenetic analysis with the Silva database release 102 (Pruesse et al., 2007; Quast et al., 2013) in the ARB software package (Ludwig et al., 2004). The initial MDA products were reamplified with the illustra GenomiPhi HY DNA Amplification kit (GE Healthcare Life Sciences, Pittsburgh, PA), using 0.5 μL of the 1:10 diluted initial amplicons in a 10 μL final reaction volume.

### Genome assembly and annotation

At the High Throughput Sequencing Facility of the University of North Carolina at Chapel Hill, six Nextera XT libraries were sequenced in multiplex with the Illumina MiSeq technology (paired-end, read length 300 bp). The genome assembly was conducted using the MetAMOS pipeline (v1.5rc3; Treangen et al., 2013). After trimming and quality filtering using EA-UTILS (Aronesty, 2013), all remaining reads were assembled using the SPades assembler with default parameters (v3.0; Bankevich et al., 2012). Genome completeness and contamination estimates based on the presence of single-copy genes was assessed using CheckM (v1.0.5; Parks et al., 2015). Detection and removal of possible contaminant contigs within the assemblies was performed in Metawatt (v3.5.2; Strous et al., 2012). All assemblies were annotated by Rapid Annotation using Subsystem Technology (RAST) in the prokaryotic annotation server (Aziz et al., 2008; Brettin et al., 2015), and checked manually by similarity searches against the sequence databases NCBI-nr, Swiss-Prot, KEGG, COG, and the protein family databases Pfam (release 27), and Inter-Pro (release 42).

### Comparison of genome assembly products

The comparison of the assemblies of each one of the segments and the merged consensus assembly was conducted using the QUality ASsessment Tool for genome assembly (QUAST v3.2; Gurevich et al., 2013). BLAST Ring Image Generator (BRIG) was used for ring visualization (Alikhan et al., 2011). Identification of single nucleotide polymorphisms (SNPs) and intrasegmental sequence heterogeneity (ISH) were conducted using QUAST and Geneious R9 (v9.0.4; Kearse et al., 2012). Following guidelines in the Illumina technical note, heterogeneity among reads is only robust in regions of coverage >30, therefore ISH determination was done only in regions with coverage ≥100, and a minimum variety frequency of 0.25. Average nucleotide identity (ANI) was estimated using Kostas ANI calculator (http://enve-omics.ce.gatech.edu/ani/) and calculations based on Goris et al. (2007).

### Accession numbers

The “*Candidatus* Marithrix sp.” sequencing project was registered under BioProject number PRJNA322859 and the individual filament segments were registered under BioSample numbers SAMN05176298 (segment 1), SAMN05176313 (segment 2), SAMN05176314 (segment 3), SAMN05176315 (segment 4), SAMN05176327 (segment 5), and SAMN05176426 (segment 6). The raw libraries were submitted to the Sequence Read Archive and the final assemblies (filtered and binned) were submitted to the Whole Genome Shotgun database. For easier access, the 16S rRNA gene sequence of the filament (100% identical across the six segments) is available under accession number KU942607 in the EMBL/EBI/DDBJ databases.

### Geochemistry analysis

Core 4562-9 of the series was collected within the mat for pore water geochemical analysis. Fixation and analysis of pore water for quantifying dissolved inorganic carbon (DIC), dissolved organic carbon (DOC), oxidized nitrogen compounds (NO_x_), orthophosphate (${\text{PO}}_{4}^{3-}$), total salinity (PSU), sulfide (H_2_S), and pH followed previously described methods (all other measurements according to Joye et al., 2004; DIC according to Brandes, 2009). Dissolved ammonium (${\text{NH}}_{4}^{+}$) was calculated from total dissolved nitrogen corrected for measured NO_x_ concentrations.

## Results and discussion

### Geochemistry of the mud volcano site and description of the “*Candidatus* Marithrix” mats

**Figure 2:**
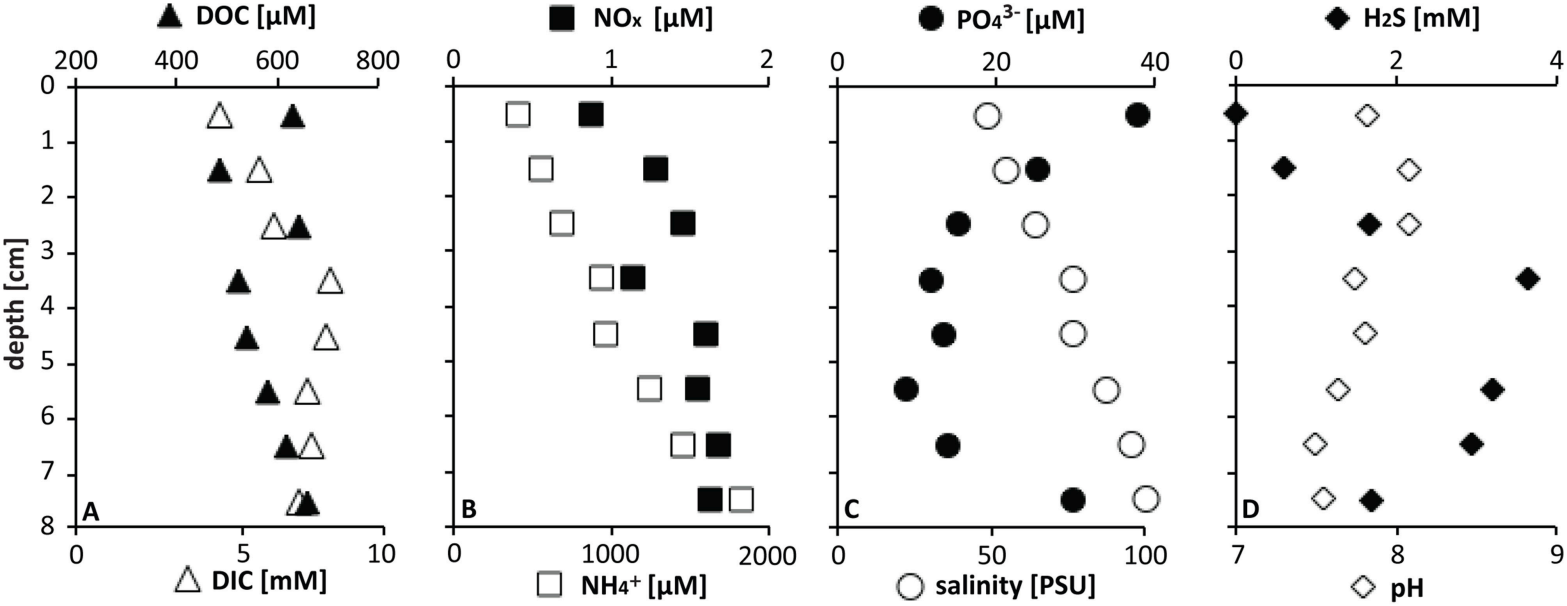
**Pore water geochemistry at GC246**.

At the mat sampling site in GC246, the downcore increasing porewater concentrations of ${\text{NH}}_{4}^{+}$, DIC, H_2_S, as well as total salinity (PSU; Figure 2) were in the typical range for seep sediments in the Gulf of Mexico (Joye et al., 2010; Lloyd et al., 2010; McKay et al., 2012), and indicate the influence of brine fluids in these sediment layers. The DOC concentrations remained about an order of magnitude lower than observed in other Gulf of Mexico briny seep cores (Joye et al., 2010). NO_x_ concentrations were generally low in reducing Gulf of Mexico seep sediments (Bowles et al., 2016). Sulfide concentrations peaked at about a depth of 4 cm and indicated sulfate reduction in near-surface sediments, as observed in other seep sites in the Gulf of Mexico (Joye et al., 2010; Lloyd et al., 2010). The mat-covered sediments at GC246 resembled the sulfide-rich, sulfate-reducing sediments of the Northwest Crater area of Mississippi Canyon Block 118 (MC118) that is covered with tufts of white sulfur bacteria very similar to those observed here (Figure 1 in Lloyd et al., 2010). The extensive mat of sulfide-oxidizing bacteria at the surface of the GC246 sediments most likely contributed to the rapidly decreasing sulfide concentrations—by approximately one millimole per centimeter—in the surficial sediment, similar to previous observations in hydrothermal vent sediments (McKay et al., 2012). Interestingly, the concentrations of orthophosphate (${\text{PO}}_{4}^{3-}$) showed a conspicuous peak at the sediment surface, suggestive of localized phosphate accumulation, as observed previously for large sulfide-oxidizing bacteria (Schulz and Schulz, 2005; Brock, 2011; Brock et al., 2012).

**Figure 3:**
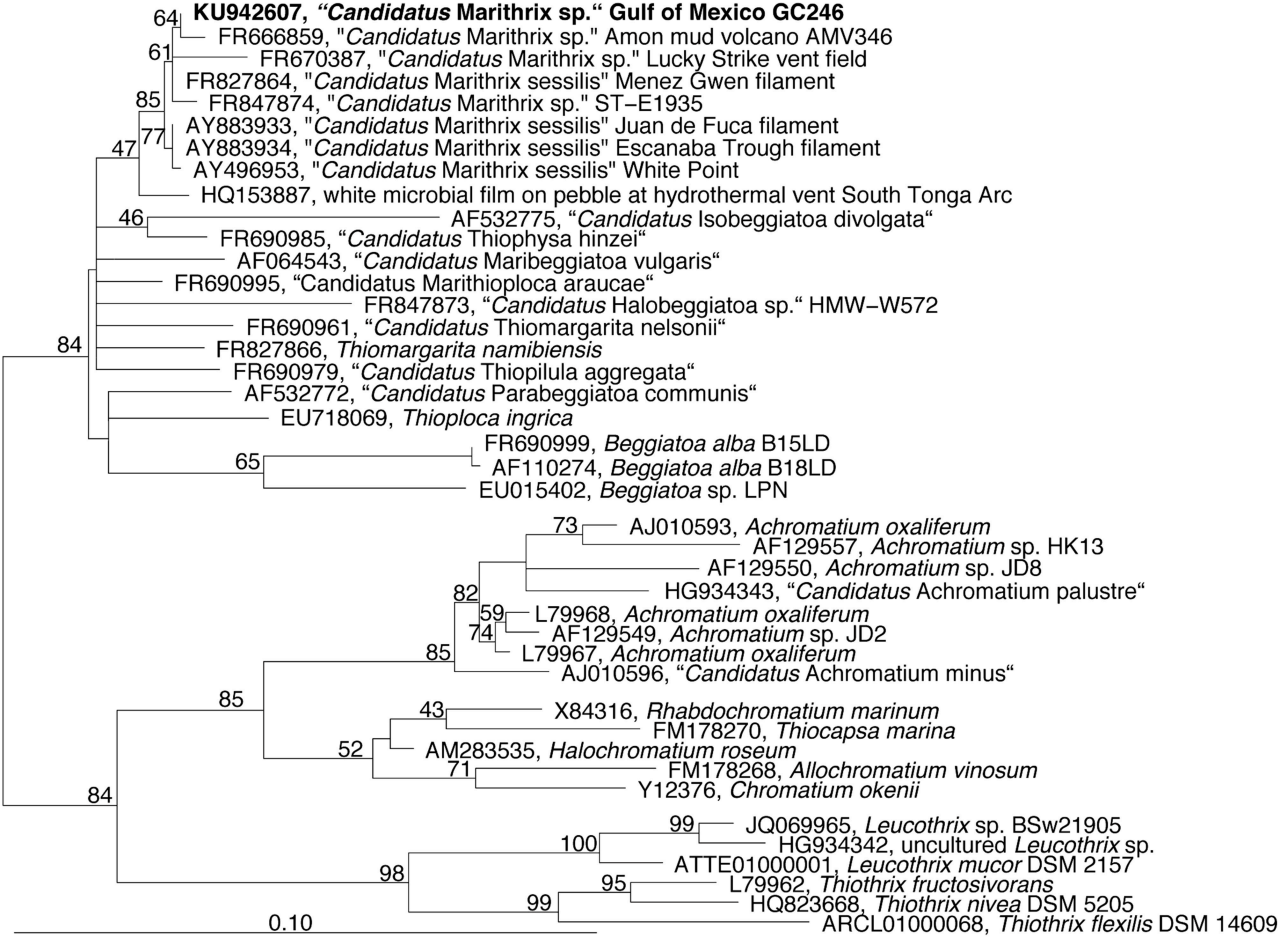
**Phylogenetic tree reconstructed from nearly full-length (***Escherichia coli*** positions 252-1313) 16S rRNA gene sequences using RaxML and a 70% positional frequency filter**. The 16S rRNA gene sequence of the Gulf of Mexico filament is shown in boldface and affiliates to other “*Candidatus* Marithrix” sequences, i.e., large attached filaments collected from vent, seep, and volcano sites across the world's oceans.

Microscopic inspection of the fluffy microbial mats at GC246 revealed that they were dominated by non-motile filaments of about 110 μm width that contained multiple sulfur globules around the periphery of the disk-shaped cells (Figure 1C). The filament diameters matched the upper limit of the previously suggested range for vacuolated, attached filaments (Kalanetra and Nelson, 2010). The interior of the cells appeared transparent, i.e., sulfur-free, and were supposedly filled by a large internal vacuole as in other large, colorless sulfur bacteria (Schulz and Jørgensen, 2001). Instead of showing the typical semi-circular ends, some terminal cells of the investigated filaments exhibited an altered morphology (Figure 1D), and were occasionally covered by sediment debris (Figure 1E). This could be indicative of a hold-fast structure, as shown for attached-living *Thiothrix* (Williams et al., 1987), *Leucothrix* (Harold and Stanier, 1955), and *Thiomargarita* (Bailey et al., 2011). The 16S rRNA gene sequence of each filament segment was 100% identical and affiliated with the other “*Candidatus* Marithrix” sequences (Figure 3). This phylogenetic cluster forms a monophyletic group within the family of large vacuolated sulfur bacteria, the *Beggiatoaceae* (Teske and Salman, 2014). The rRNA genes of the “*Candidatus* Marithrix” filament do not contain any introns, as described for some of the other members of the family (Salman et al., 2012; MacGregor et al., 2013b; Flood et al., 2016; Winkel et al., 2016).

### Recovery of nearly complete genomic datasets for each individual filament segment

A single “*Candidatus* Marithrix” filament was manually separated into six equal segments of roughly 30 cells that were sequenced independently. Consistently, each segment library contained between 2.6 and 2.9 million pairs of reads, except for segment 4 that contained only 1.5 million pairs of reads (Table 1). Initially, in segments 1–4 we were able to reconstruct roughly 3.6 Mbp of genomic data for each segment with a number of contigs between 220 and 395. The assemblies of segments 5 and 6 were much more fragmented (>1000 and 790 contigs, respectively), and segment 5 assembled into a slightly larger dataset of 4.4 Mbp. Completeness in all assemblies was consistently ~93%. However, contamination estimates based on the presence of single-copy genes were occasionally above 6%, which is considered above the error range estimated for incomplete (~70%) genomes (Parks et al., 2015). To reduce contamination, we conducted sequence binning of the assemblies using Metawatt, based on coverage, tetranucleotide patterns, and taxonomic assignment. This step drastically reduced the number of contigs in each assembly, and increased the N50 from about 66 to 82 kbp (Table 1), suggesting that the majority of short contigs were removed. In terms of the assembly length, this step removed about 10% of sequence length for each of the segments 1–4 that still contained ca. 3.2 Mbp afterwards. Segments 5 and 6 lost 42 and 34% of their total sequence length in the binning step, yielding ca. 2.5 Mbp assembly lengths each. For all datasets, this step reduced the contamination percentage to 1–4%, and the reassessed completeness of the assemblies remained around 90% (Table 1).

**Table 1:** **Comparison of the datasets of the six individually sequenced filament segments**.

	**Segment 1**	**Segment 2**	**Segment 3**	**Segment 4**	**Segment 5**	**Segment 6**
Library size [read pairs]	2,815,883	2,613,483	2,723,839	1,511,409	2,870,441	2,937,079
Library size after qc [read pairs]	2,812,811	2,609,986	2,711,518	1,510,768	2,868,622	2,935,885
**AFTER METAMOS**						
Assembly length [bp]	3,587,597	3,692,126	3,517,120	3,568,359	4,417,874	3,621,759
# Contigs	292	395	220	315	1153	790
N50 [bp]	95,376	76,817	118,717	83,432	13,383	11,451
G+C [%]	34.59	35	34.4	34.31	35.06	34.79
Completeness [%]	93.75	93.75	93.75	93.75	93.19	93.19
Contamination [%]	4.78	5.78	4.75	7.49	11.88	5.48
**AFTER METAWATT**						
Assembly length [bp]	3,210,343	3,217,969	3,239,714	3,116,424	2,544,604	2,402,354
# Contigs	63	66	44	71	215	252
N50 [bp]	101,092	94,039	166,492	94,368	20,973	16,036
G+C [%]	34.02	34.03	34.04	34.03	34.13	34.09
Completeness [%]	93.75	93.19	93.75	93.75	90.85	89.17
Contamination [%]	3.6	4.17	3.04	2.48	2.57	1.12
**FINAL CHARACTERISTICS**						
Coverage	409	362	398	218	468	444
Reads mapped	4,379,585	3,887,216	4,293,393	2,264,295	3,969,380	3,555,212
Total CDS	3350	3334	3359	3224	2580	2420
Hypothetical genes	1286	1272	1278	1211	896	826
rRNA genes	3	3	3	3	3	0
tRNA genes	39	39	39	39	38	33
bp loss after Metawatt [%]	0.11	0.13	0.08	0.13	0.42	0.34

*The original data were passed through the MetAMOS pipeline and were further filtered in Metawatt to create the final datasets. CDS, coding DNA sequences*.

Even though the individual segments yielded highly complete genomes, we attempted to assemble a consensus genome using all six libraries. This approach produced an extremely fragmented assembly with more than 10,000 contigs; a possible consequence of introducing contaminant reads from some of the individual libraries (see below). Therefore, in a second attempt we reduced the size of the combined libraries by using only those reads that mapped to the final binned assembly of each segment. Yet again, the combined assembly could not be improved compared to the individual assemblies: despite slightly higher total length (3.3 Mbp) the dataset was much more fragmented (509 contigs) with lower N50 (18,534); the genome completeness remained at 93% with a fairly high contamination level of 4.35%.

Previous findings suggested that MDA introduces a bias, amplifying preferred regions while underrepresenting others (Raghunathan et al., 2005; Lasken, 2007); in particular, high G+C regions are discriminated against (Pinard et al., 2006; Yilmaz et al., 2010). Accordingly, it can be speculated that the MDA reaction with the “*Candidatus* Marithrix” segments did not cover all regions of the genomes, and thus a small but consistent genome fraction could be missing. Initial studies with the MDA technique and the 454 Life Science sequencing platform reported that a genome completeness of 70–75% could be expected at best (Lasken, 2007). Greater sequencing depth should result in a more complete dataset (Lasken, 2007), and indeed Illumina MiSeq achieved 93% here. Yet, our attempt to artificially increase the library size by combining multiple clonal libraries did not produce a more complete genome. Even though the missing regions that prevent the closing of the “*Candidatus* Marithrix” genome cannot be identified at this point, we doubt the role of MDA bias as the cause, and instead suggest that ambiguous sequences such as repetitive elements cause discrepancies during the assembly process. When repetitive regions collapse during assembly, correct positioning and identification of flanking regions becomes problematic. We identified hotspots with an increased coverage signature (>1000x)—an aberration of the 380x mean coverage—that could be indicative of such repetitive elements. These regions of strong read-overrepresentation are located on some long contigs as well as on the entire length of some shorter contigs, and could hinder full genome assembly. However, a recent study showed that it is not always necessary to close a genome to capture the full functional potential of an organism (Fadeev et al., 2016). Also, our intended analysis of genome plasticity is not affected by the closing status of the datasets. Therefore, we continue our analysis with the six individual segment datasets, of which segments 1–4 resembled each other strongly in terms of completeness, contamination, and fragmentation (Table 1).

During Metawatt binning, each dataset of the six segments revealed a number of contigs that were short, with low coverage, and that were removed from the final datasets. In segments 5 and 6, additional contigs with longer N50 and higher coverage were binned out. Taxonomically, these contigs affiliated to several other groups of microorganisms that are typically found in the marine environment (Table S1). As a consequence, the final assemblies for segments 5 and 6 are less complete (Table 1). It is conspicuous that toward this one end of the filament, the amount and phylogenetic diversity of contaminating contigs became more pronounced although preparation and handling was the same for all segments. We hypothesize that the source organisms of these contigs (in datasets 5 and 6) were actually associated with the filament segment, and might represent epibionts or bacteria caught in a polysaccharide sheath or recalcitrant exopolymer layer that was more resistant to manual removal. It is possible that this end of the filament might have been in closer proximity to the holdfast end that could have been lined with a more pronounced organic matrix.

### Genomes are highly conserved along the filament, while increased ISH reflects polyploidy

**Figure 4:**
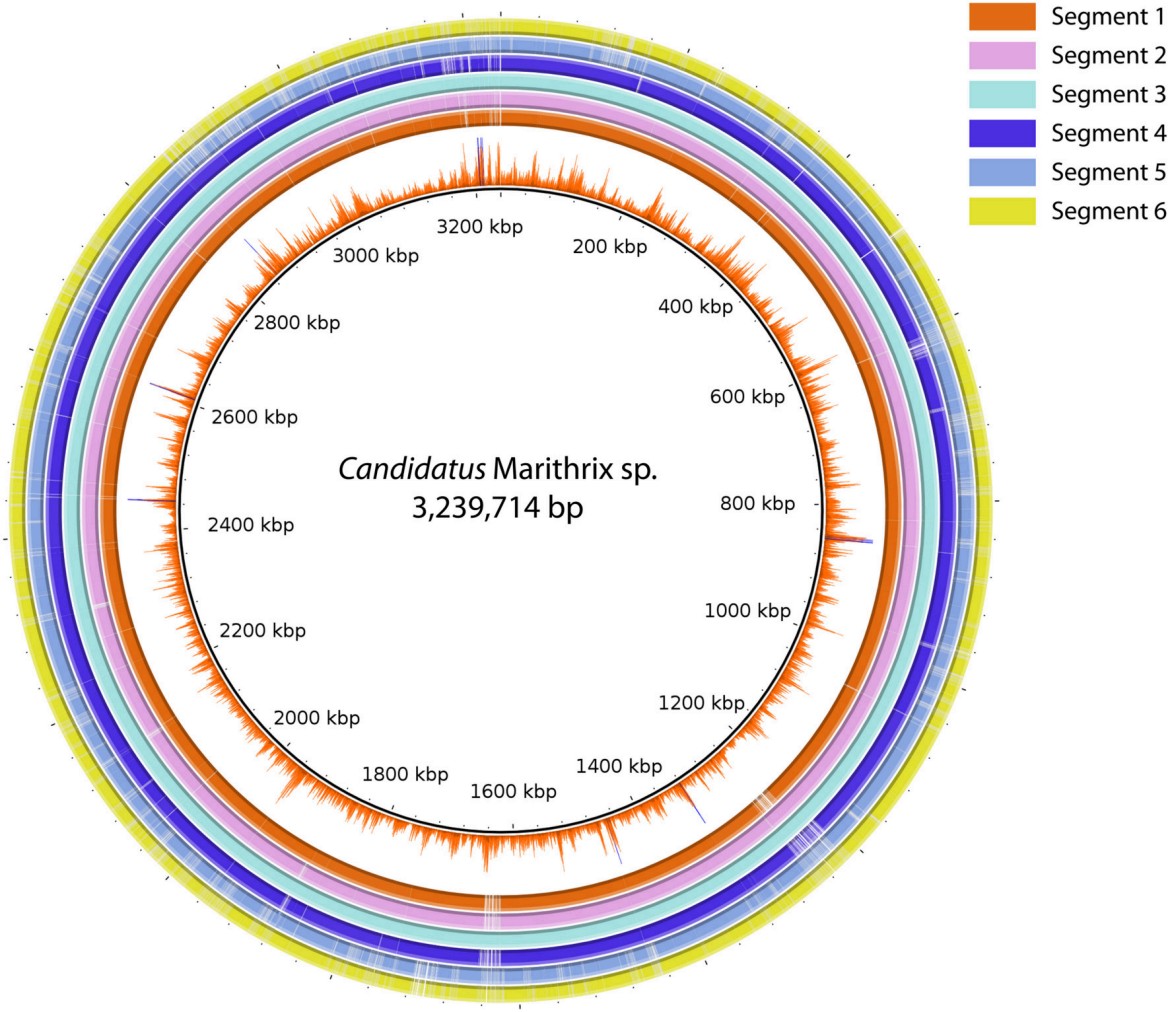
**Sequence comparison between all six segment datasets using BLASTN ring image generator**. Segment 3 was used as reference due to the most successful assembly result. The inner-most ring represents the Illumina coverage of the segment 3 assembly. All regions in the figure represent at least 70% identity and an *e*-value ≤ 0.000001. The majority of sequence information is represented in all six datasets, and only segment 5 and 6 reveal larger regions with gaps, i.e., missing information in comparison to the other assemblies.

We were able to recover about 90% of the “*Candidatus* Marithrix” genome from each filament segment, and the genomic information is to the highest extent redundant. Two-way calculated average nucleotide identities (ANI) among the six assemblies ranged between 99.98–100% (Table S2). The genome assembly of segment 3 represented the longest and most complete of all six assemblies (Table 1), and was therefore selected as reference genome. BlastN alignment of all assemblies to the reference showed that nearly all of the recovered genomic information is represented across all datasets (Figure 4). Functional plasticity among the datasets was assessed by comparing gene representation based on the SEED subsystems, which revealed a strong overlap in functional genes representation between the segments (Table S3) —in some categories even all six segments contained exactly the same amount of genes. Furthermore, a sequence-based comparison showed that segment 3 contributed most to new sequence data; these regions are occasionally long enough to have a potential assigned function (Tables S4, S5). Each of the other assemblies contained only short unique sequence regions that represent mainly truncated or hypothetical genes (Table S4). Function-based comparison revealed likewise few (5 or less) unique functional entities in each dataset (Table S5).

One of the hypotheses of this study was that cells/segments with increased distance to each other also show an increased separation on the genetic level. Therefore, we investigated the frequency of SNPs along the artificial segments of the filament. The comparison of all assemblies to each other revealed an average ratio of 4.2 ± 1 SNPs/100 kbp among segments 1–4. However, a directionality of increasing or decreasing SNPs could not be observed (Table 2). Previous studies report SNPs anywhere between <10 and several 100 per genome (Holt et al., 2008; Harris et al., 2010; Limmathurotsakul et al., 2014), so this number is well within the range. Segments 5 and 6 had an increased amount of SNPs among each other and to all other segments (19.2 ± 7.1 SNPs/100 kbp). We speculate that this increased heterogeneity is based on the lower quality of the sequencing process of segments 5 and 6, because roughly 40% of the data originated from non-target DNA (Table S1). Therefore, we treat the analysis of heterogeneity in segments 5 and 6 with more caution and focus on segments 1–4 only. Searching for SNPs in the coding regions of the assemblies revealed that the average ratio slightly decreased to 3.6 ± 0.7 SNPs/100 kbp. In non-coding regions, the ratio of SNPs doubled to 8.5 ± 6.5/100 kbp (Table 2), revealing that the majority of SNPs are located in the non-coding regions. The locations of most indels (insertion or deletion of single nucleotides) were also more abundant in non-coding regions as the average ratio even dropped from 0.6 ± 0.2 indels/100 kbp in the full assemblies to 0.2 ± 0.1/100 kbp in the coding regions, and increased substantially to 3.9 ± 1.3/100 kbp in non-coding regions (Table 2). We conclude that indels are generally found with lesser frequency in coding regions because they corrupt the reading frame and thus create pseudogenes, while SNPs may be found here at a higher frequency than indels because a SNP retains the reading frame, and can either represent a silent mutation, or cause at most a usage of a different amino acid. As with SNPs, the frequency of indels did not correlate with physical proximity of segments to each other (Table 2).

**Table 2:** **Comparison of heterogeneity among the contigs of the six datasets**.

	**Segment 1**	**Segment 2**	**Segment 3**	**Segment 4**	**Segment 5**	**Segment 6**
Assembly length [bp]	3,210,343	3,217,969	3,239,714	3,116,424	2,544,604	2,402,354
CDS [bp]	2,836,815	2,843,091	2,882,289	2,757,582	2,221,023	2,106,546
nCDS [bp]	373,528	374,878	357,425	358,842	323,581	295,808
Coding region [%]	88.36	88.35	88.97	88.49	87.28	87.69
**SEGMENT 1**						
Total SNPs	0	132	86	145	300	757
SNPs/100 kbp	0	4.10	2.65	4.65	11.79	31.51
Total indels	0	8	14	27	41	82
Indels/100 kbp	0	0.25	0.43	0.87	1.61	3.41
Total SNPs in CDS	0	91	66	124	136	316
SNPs in CDS/100 kbp	0	3.20	2.29	4.50	6.12	15.00
SNPs in nCDS/100 kbp	0	10.94	5.60	5.85	50.68	149.08
Total indels in CDS	0	3	0	8	12	15
Indels in CDS/100 kbp	0	0.12	0	0.34	0.63	0.82
Indels in nCDS/100 kbp	0	1.33	3.92	5.29	8.96	22.65
**SEGMENT 2**						
Total SNPs	151	0	132	135	302	682
SNPs/100 kbp	4.70	0	4.07	4.33	11.87	28.39
Total indels	16	0	15	17	44	64
Indels/100 kbp	0.50	0	0.46	0.55	1.73	2.66
Total SNPs in CDS	94	0	116	135	138	303
SNPs in CDS/100 kbp	3.31	0	4.02	4.90	6.21	14.38
SNPs in nCDS/100 kbp	15.26	0	4.48	0	50.68	128.12
Total indels in CDS	3	0	3	3	12	13
Indels in CDS/100 kbp	0.11	0	0.10	0.11	0.54	0.62
Indels in nCDS/100 kbp	3.48	0	3.36	3.90	9.89	17.24
**SEGMENT 3**						
Total SNPs	84	118	0	194	341	798
SNPs/100 kbp	2.62	3.67	0	6.23	13.40	33.22
Total indels	24	15	0	24	47	76
Indels/100 kbp	0.75	0.47	0	0.77	1.85	3.16
Total SNPs in CDS	84	101	0	118	140	325
SNPs in CDS/100 kbp	2.96	3.55	0	4.28	6.30	15.43
SNPs in nCDS/100 kb	0.00	4.53	0	21.18	62.12	159.90
Total indels in CDS	0	3	0	8	12	15
Indels in CDS/100 kbp	0	0.11	0	0.29	0.54	0.71
Indels in nCDS/100 kbp	6.43	3.20	0	4.46	10.82	20.62
**SEGMENT 4**						
Total SNPs	126	153	146	0	358	568
SNPs/100 kbp	3.92	4.75	4.51	0	14.07	23.64
Total indels	24	13	24	0	43	64
Indels/100 kbp	0.75	0.40	0.74	0	1.69	2.66
Total SNPs in CDS	91	94	112	0	158	301
SNPs in CDS/100 kbp	3.21	3.31	3.89	0	7.11	14.29
SNPs in nCDS/100 kbp	9.37	15.74	9.51	0	61.81	90.26
Total indels in CDS	8	3	8	0	21	13
Indels in CDS/100 kbp	0.28	0.11	0.28	0	0.95	0.62
Indels in nCDS 100 kbp	4.28	2.67	4.48	0	6.80	17.24
**SEGMENT 5**						
Total SNPs	374	352	394	456	0	548
SNPs/100 kbp	11.65	10.94	12.16	14.63	0	22.81
Total indels	55	54	61	59	0	58
Indels/100 kbp	1.71	1.68	1.88	1.89	0	2.41
Total SNPs in CDS	192	162	153	209	0	201
SNPs in CDS/100 kbp	6.77	5.70	5.31	7.58	0	9.54
SNPs in nCDS/100 kbp	48.72	50.68	67.43	68.83	0	117.31
Total indels in CDS	17	17	17	26	0	16
Indels in CDS/100 kbp	0.60	0.60	0.59	0.94	0	0.76
Indels in nCDS/100 kbp	10.17	9.87	12.31	9.20	0	14.20
**SEGMENT 6**						
Total SNPs	663	624	712	649	587	0
SNPs/100 kbp	20.65	19.39	21.98	20.83	23.07	0
Total indels	80	75	76	77	65	0
Indels/100 kbp	2.49	2.33	2.35	2.47	2.55	0
Total SNPs in CDS	354	339	342	345	193	0
SNPs in CDS/100 kbp	12.48	11.92	11.87	12.51	8.69	0
SNPs in nCDS/100 kbp	82.72	76.02	103.52	84.72	121.76	0
Total indels in CDS	15	13	15	15	16	0
Indels in CDS/100 kbp	0.53	0.46	0.52	0.54	0.72	0
Indels in nCDS/100 kbp	17.40	16.54	17.07	17.28	15.14	0

*As all datasets have a different length, total numbers of mismatches are also given as a ratio per 100 kb based on aligned regions. Single nucleotide polymorphisms (“SNPs”) represent a single exchange of a nucleotide; “indel” represents an insertion or deletion of a single nucleotide; CDS, coding DNA sequence; nCDS, non-coding DNA sequence*.

In order to extend the analysis, we investigated the read heterogeneity within each segment library. Each assembly represents a genomic consensus of about 30 cells, of which each cell has an unknown level of polyploidy. The reconstructed genome of one filament segment thus represents a metagenome of these multiple, polyploid cells. Assuming similar polyploidy levels as in *Epulopiscium*, a similarly large (>100 μm) polyploid bacterium, a single cell can have tens of thousands, or even hundreds of thousands, of genome copies that are highly identical (Mendell et al., 2008). The coverage of our dataset is not nearly sufficient to capture all possible genome varieties per cell, and no currently available sequencing technique can achieve this. Nevertheless, on a relative level, we can assess at which level the libraries of each segment reveal sequence polymorphisms. As this analysis would represent the heterogeneity among genome copies within a segment, we propose the term intrasegmental sequence heterogeneity (ISH). We used the assembly of segment 3 as the framework sequence and mapped each of the six Illumina libraries to it. The variants in the libraries were determined only in regions with coverage of ≥100x, and with a variety frequency of ≥0.25. The results revealed an average of 2261 ISH events (single nucleotide variants and indels) in each library (Table S6), 740 of which are present in all six segments. Accordingly, a high degree of conservation among the genome copies, as suggested in *Epulopiscium* (Mendell et al., 2008), can also be found in polyploid “*Candidatus* Marithrix” cells. About a third of the ISH events are not randomly distributed, but are re-occurring at specific sites among the genome copies of all filament segments. Two thirds of the ISH events, however, are not co-localized, and thus represent the unique case of genetic heterogeneity across multiple genome copies as it can only occur in a polyploid cell. We speculate that these positions represent hotspots for genome plasticity, and possibly microevolution. Polyploidy and extensive distribution of DNA across a large cell body as in “*Candidatus* Marithrix,” *Thiomargarita*, or *Epulopiscium* (Angert, 2012) suggests functional compartmentalization as an adaptation to a large cell body (Mendell et al., 2008), but remains to be shown for any of these large, highly polyploid bacteria.

Our intra- and inter-genomic analyses show that the amount of SNPs in the collapsed genome assemblies is at least an order of magnitude lower than the ISH events among multiple genome copies (compare Table 2 “total SNPs,” and Table S6). Since current sequencing technologies cannot cover all varieties in the supposedly >10,000x polyploid cells, the evaluation of ISH events is most likely far underestimated. However, regardless of the high polyploidy and large potential for genome heterogeneity in these large bacteria, the consensus of genetic information, especially in the coding regions, across a few hundred clonal cells is highly conserved. Furthermore, finding equal levels of ISH along the filament segments, and their frequent occurrence at identical genome positions even suggests that genome copies may be separated proportionally during cell division, contributing to the high level of genomic conservation. We can only speculate about the division process of “*Candidatus* Marithrix,” i.e., while binary fission has been observed (Kalanetra et al., 2004; Kalanetra and Nelson, 2010) there is no information about the age of cells along the filament. In the likewise sessile, filamentous, *Thiothrix* spp. no directionality of cell division has been reported, which means that any cell along the filament is capable to divide at any given time. With this in mind, our analysis of genetic diversification could also not resolve whether those cells with closer physical proximity are more recent (younger) offspring to each other than cells with increased distance.

### Insights to the ecophysiology of “*Candidatus* Marithrix” revealed by the genome

The assembly of segment 3 was chosen as reference for the “*Candidatus* Marithrix” filament genome analysis because it represents the longest sequence assembly with the lowest number of contigs, and a low level of contamination. The analysis reveals that “*Candidatus* Marithrix” is a typical facultative aerobic sulfide-oxidizer, but that it lacks genes for motility/chemotaxis. Thus, *Candidatus* Marithrix” shares many characteristics with other large sulfur-oxidizing bacteria of the *Beggiatoaceae*, with the conspicuous exception of its non-motile, sessile lifestyle.

The genome encodes several genes assisting during oxidative stress, which is of ecological relevance because the filaments inhabit the sediment surface. Here, oxygen penetrates typically 1–2 mm before it is depleted (de Beer et al., 2006; Grünke et al., 2012). Encoded proteins that could protect “*Candidatus* Marithrix” against oxidation damage by radicals are the iron-containing superoxide dismutase, glutaredoxins, rubrerythrin, as well as the NnrS protein involved in response to NO. While this arsenal suggests that “*Candidatus* Marithrix” can withstand higher oxygen concentrations, we also hypothesize that the organism respires oxygen as an electron acceptor. The genome encodes the complexes of the electron transport chain for oxidative phosphorylation, including a cbb3-cytochrome c terminal oxidase.

So far, vacuolar nitrate accumulation as observed in other large sulfide-oxidizing bacteria could not be detected in “*Candidatus* Marithrix” (Kalanetra et al., 2004; Kalanetra and Nelson, 2010). However, the nitrate content of the vacuoles is highly dynamic and probably reflects the nitrate access and availability in the surrounding (pore) water. In this “*Candidatus* Marithrix” genome, we find several genes encoding proteins for nitrate respiration as observed in other nitrate-accumulating vacuolated bacteria (Mussmann et al., 2007; MacGregor et al., 2013a). One operon encodes successively the following genes: nitrate/nitrite response regulator, nitrate/nitrite sensor protein, two nitrate/nitrite transporters, and the four subunits of the respiratory, membrane-bound nitrate reductase (*narGHIJ*) performing the first reductive step in denitrification. Furthermore, the periplasmatic nitrate/nitrite reductase is present with the large and small subunits (*napAB*); this enzyme functions in denitrification as well as dissimilatory nitrate reduction to ammonia (DNRA). The *NasAB* subunits are present as well and function in nitrate/nitrate assimilation for ammonification. We further identify the gene for nitrite reductase (*nirS*)/cytochrome cd1 nitrite reductase, forming NO from nitrite. However, the reconstruction of the denitrification pathway cannot be completed, because none of the nitric oxide reductase subunits (*norBC*), nor the nitrous oxide reductase (*nosZ*) were identified with great confidence. The NAD(P)H-nitrite reductase (*nirBD*) of the DNRA pathway, however, was present with both subunits. While these results support the working hypothesis that “*Candidatus* Marithrix” can respire nitrate to some extent, the apparent pathway gaps prevent identifying the end product of nitrate reduction. There is no genomic evidence that “*Candidatus* Marithrix” is capable of storing nitrate in vacuoles, or for the vacuolar-type ATP hydrolase reported for vacuolated relatives of “*Candidatus* Marithrix” (Mussmann et al., 2007; Winkel et al., 2016).

We can reconstruct a complete sulfide oxidation pathway, starting with two enzymes for the initial step of sulfide oxidation. The genome contains the gene for a sulfide:quinone oxidoreductase (*sqr*, FAD-dependent pyridine nucleotide-disulphide oxidoreductase), and both subunits of the alternative sulfide oxidation enzyme flavocytochrome c sulfide dehydrogenase in one operon (*fccAB*). For the oxidation of thiosulfate to elemental sulfur, we identify the genes *soxBXYZ*, while the genome lacks the *soxCD* genes, as reported for sulfur-accumulating bacteria (Dahl and Prange, 2006; Dahl and Friedrich, 2008). For the final oxidation of the intermediate zero-valent sulfur (polysulfide and/or elemental sulfur), the “*Candidatus* Marithrix” genome encodes the genes for the reverse dissimilatory sulfite reductase pathway (*dsrABGJKMOPR*), as well as both subunits for the adenylylsulfate reductase (*aprAB*), and the sulfate adenylyltransferase (*sat*).

The presence of the large and small subunit of the ribulose bisphosphate carboxylase indicates that “*Candidatus* Marithrix” contains a form I RubisCO for carbon fixation. Interestingly, the genome also contains multiple copies of the high-affinity carbon uptake Hat/HatR protein with some copies being concatenated on the same contig. This protein is supposedly involved in CO_2_ uptake into carboxysomes in cyanobacteria. Experimental evidence shows that RubisCO activity is detectable in “*Candidatus* Marithrix,” fixing ca. 2.5 nmol CO_2_ min^−1^ mg^−1^ protein (Kalanetra and Nelson, 2010), which however is much less compared with rates determined in a *Beggiatoa* culture with >70 nmol CO_2_ fixed min^−1^ mg^−1^ protein (Nelson and Jannasch, 1983). Besides the lithotrophic sulfide oxidation pathway, the “*Candidatus* Marithrix” genome reveals the capability to use organic compounds for energy generation. Besides the genes for glycolysis and the TCA cycle we find a glycogen synthase and phosphorylase, indicating that the carbon polymer glycogen may be a storage compound not only in *Thiomargarita* (Schulz and Schulz, 2005), but also in “*Candidatus* Marithrix.”

## Conclusion

The first genomic sequence for the large, vacuolated, marine, attached-living “*Candidatus* Marithrix” reveal the usage of reduced sulfur, and organic compounds, as well as oxygen and nitrate for energy generation. Lithoautotrophy can be inferred from the presence of key carbon fixation-related genes. Hence, “*Candidatus* Marithrix” shares genetic capacities that are typical for sediment-dwelling gradient organisms; the pathways identified here are consistent with reported characteristics of their relatives in the *Beggiatoaceae*. The main focus of this study, however, was the degree of genome conservation across manually separated segments of a single filament of “*Candidatus* Marithrix.” We showed that the individual assemblies of filament segments demonstrate a very high level of conservation, while each assembly represents about 90% of the genome. The assembly process collapses ambiguous regions such as repetitive elements, and thus hinders the closing of a consensus genome. The relatively moderate amount of SNPs across the assemblies indicates a high level of genome conservation, i.e., a high level of sequence and functional redundancy across the multiple genome copies of the filament. However, the strongly increased heterogeneity per position in the libraries shows that the genome copies have a much greater variation within than across different segments of a filament. Yet again, the frequent co-localization of these variants across the filament segments indicates that genetic information remains highly conserved. However, variant positions also have an increased potential for representing hotspots of microevolution within a polyploid cell, and therefore represent hotspots of genome plasticity for a filament, analogous to a clonal microcolony of cells.

In our study, we were not only limited in currently available sequencing techniques to sufficiently cover all potential heterogeneity of polyploidy. Also, a single filament only provides genetic information of a few hundred generations of clonal cells, which might not yield sufficient resolution to reveal the progression of genomic diversification. Furthermore, we lack information about generation times of “*Candidatus* Marithrix” organisms, or about the evolutionary pressures on genome conservation. Following indications for their better-studied *Thiomargarita* relatives both long generation times and low evolutionary pressure can be assumed; *Thiomargarita* cells are suggested to survive for months in anaerobic sediments using intracellularly stored nitrate as electron acceptor, and even after 2 years live populations were still found in *ex situ* stored sediments (Schulz et al., 1999). In this light, our findings of high genome conservation support the hypothesis that evolution and differentiation in “*Candidatus* Marithrix” and related large sulfur-oxidizing bacteria require very long time scales.

Even though we cannot detect much genetic difference or genomic evolution along the filament, we cannot exclude that individual cells differ in their expression, i.e., functional compartmentalization. This information, though, would contribute majorly to the fundamental understanding of the functionality of filaments, and the interplay of cells therein. As reported for other filamentous organisms, cells can differentiate morphologically and physiologically, e.g., cyanobacteria forming heterocysts (Sandh et al., 2009), or may retain their morphology while their functionality changes (Sheik et al., 2016). Functional compartmentalization has also been suggested for other “regular-sized” bacteria (Cornejo et al., 2014), and has been experimentally shown in sporulating *Bacillus* sp. (Hilbert and Piggot, 2004). For future studies, we thus propose to include analyses that can shed some light on the gene expression in individual cells, or their specific phenotypic activities. Of special interest could be the individual cellular reactivity to chemical stimuli, e.g. in the filamentous “*Candidatus* Marithrix,” or the chain-forming *Thiomargarita*, but especially for the motile, gliding *Beggiatoa*-like filaments.

## Author contributions

VS-C designed the work, conducted the DNA sequencing, data analysis, critical data interpretation, and wrote the manuscript. EF performed sequence data analysis, assisted in critical data interpretation, and in writing the manuscript. SJ provided access to specimen, conducted geochemical analysis and critical data interpretation, and assisted in writing the manuscript. AT sampled the specimen, assisted in the design of the work, assisted in critical data interpretation, and in writing the manuscript. All authors approved the final version of the manuscript.

## Funding

Cruise AT18-02 with RV Atlantis and HOV Alvin were supported by NSF grant EF-0801741 in the program “Emerging Frontiers/Microbial Observatories and Microbial Interactions and Processes” (Collaborative Research: A Microbial Observatory examining Microbial Abundance, Diversity, Associations, and Activity at Seafloor Brine Seeps) to SJ and AT. VS-C was supported by the Deutsche Forschungsgemeinschaft grant SA2505/1-1 and SA2505/2-1.

### Conflict of interest statement

The authors declare that the research was conducted in the absence of any commercial or financial relationships that could be construed as a potential conflict of interest.

## References

[B1] Alikhan N.-F. Petty N. K. Zakour N. L. B. Beatson S. A. (2011). BLAST ring image generator (BRIG): simple prokaryote genome comparisons. BMC Genomics 12:402. 10.1186/1471-2164-12-402 21824423 PMC3163573

[B2] Angert E. R. (2006). The enigmatic cytoarchitecture of *Epulopiscium* spp., in Microbiology Monographs 2: Complex Intracellular Structures in Prokaryotes, ed Shiverly J. M. (Berlin; Heidelberg: Springer), 285–301. 10.1007/7171_027

[B3] Angert E. R. (2012). DNA replication and genomic architecture of very large bacteria. Annu. Rev. Microbiol. 66, 197–212. 10.1146/annurev-micro-090110-102827 22994492

[B4] Aronesty E. (2013). Comparison of sequencing utility programs. Open Bioinformatics J. 9, 1–8. 10.2174/1875036201307010001

[B5] Aziz R. K. Bartels D. Best A. A. DeJongh M. Disz T. Edwards R. A.. (2008). The RAST server: rapid annotations using subsystems technology. BMC Genomics 9:75. 10.1186/1471-2164-9-75 18261238 PMC2265698

[B6] Bailey J. V. Salman V. Rouse G. Schulz-Vogt H. N. Levin L. Orphan V. (2011). Dimorphism in methane seep-dwelling ecotypes of the largest known bacteria. ISME J. 5, 1926–1935. 10.1038/ismej.2011.66 21697959 PMC3223306

[B7] Bankevich A. Nurk S. Antipov D. Gurevich A. A. Dvorkin M. Kulikov A. S.. (2012). SPAdes: a new genome assembly algorithm and its aplications to single-cell sequencing. J. Comp. Biol. 19, 455–477. 10.1089/cmb.2012.0021 22506599 PMC3342519

[B8] Bowles M. Hunter K. S. Samarkin V. Joye S. (2016). Patterns and variability in geochemical signatures and microbial activity within and between diverse cold seep habitats along the lower continental slope, Northern Gulf of Mexico. Deep Sea Res. II 129, 31–40. 10.1016/j.dsr2.2016.02.011

[B9] Brandes J. A. (2009). Rapid and precise δ13C measurement of dissolved inorganic carbon in natural waters using liquid chromatography coupled to an isotope-ratio mass spectrometer. Limnol. Oceanogr. 7, 730–739. 10.4319/lom.2009.7.730

[B10] Bresler V. Fishelson L. (2003). Polyploidy and polyteny in the gigantic eubacterium *Epulopiscium fishelsoni*. Mar. Biol. 143, 17–21. 10.1007/s00227-003-1055-2

[B11] Brettin T. Davis J. J. Disz T. Edwards R. A. Gerdes S. Olsen G. J.. (2015). RASTtk: A modular and extensible implementation of the RAST algorithm for building custom annotation pipelines and annotating batches of genomes. Sci. Rep. 5:8365. 10.1038/srep08365 25666585 PMC4322359

[B12] Brock J. (2011). Impact of Sulfide-Oxidizing Bacteria on the Phosphorus Cycle in Marine Sediments. Ph.D. thesis, University of Bremen.

[B13] Brock J. Rhiel E. Beutler M. Salman V. Schulz-Vogt H. N. (2012). Unusual polyphosphate inclusions observed in a marine *Beggiatoa* strain. Antonie Van Leeuwenhoek 101, 347–357. 10.1007/s10482-011-9640-8 21909788 PMC3261416

[B14] Cornejo E. Abreu N. Komeili A. (2014). Compartmentalization and organelle formation in bacteria. Curr. Opin. Cell Biol. 26, 132–138. 10.1016/j.ceb.2013.12.007 24440431 PMC4318566

[B15] Dahl C. Friedrich C. G. (2008). Microbial Sulfur Metabolism. Berlin Heidelberg; New York, NY: Springer.

[B16] Dahl C. Prange A. (2006). Bacterial sulfur globules: occurrence, structure and metabolism, in Microbiology Monographs 1: Inclusions in Prokaryotes, ed Shiverly J. M. (Berlin; Heidelberg: Springer), 21–51. 10.1007/7171_002

[B17] de Beer D. Sauter E. Niemann H. Kaul N. Foucher J. P. Witte U. (2006). *In situ* fluxes and zonation of microbial activity in surface sediments of the Hakon Mosby mud volcano. Limnol. Oceanogr. 51, 1315–1331. 10.4319/lo.2006.51.3.1315

[B18] Fadeev E. De Pascale F. Vezzi A. Hübner S. Aharonovich D. Sher D. (2016). Why close a bacterial genome? The plasmid of *Altermononas macleodii* HOT1A3 is a vector for inter-specific transfer of a flexible genomic island. Front. Microbiol. 7:248. 10.3389/fmicb.2016.00248 27014193 PMC4781885

[B19] Flood B. Fliss P. Jones D. S. Dick G. J. Jain S. Kaster A.-C.. (2016). Single-cell (meta-) genomics of a dimorphic “*Candidatus* Thiomargarita nelsonii” reveals genomic plasticity. Front. Microbiol. 7:603. 10.3389/fmicb.2016.00603 27199933 PMC4853749

[B20] Friedberg E. C. (2003). DNA damage and repair. Nature 421, 436–440. 10.1038/nature01408 12540918

[B21] Goris J. Konstantinidis K. T. Klapperbach J. A. Coenye T. Vandamme P. Tiedje J. M. (2007). DNA-DNA hybridization values and their relationship to whole-genome sequence similaities. Int. J. Syst. Evol. Microbiol. 57, 81–91. 10.1099/ijs.0.64483-0 17220447

[B22] Grünke S. Felden J. Lichtschlag A. Girnth A. C. De Beer D. Wenzhofer F.. (2011). Niche differentiation among mat-forming, sulfide-oxidizing bacteria at cold seeps of the Nile Deep Sea Fan (Eastern Mediterranean Sea). Geobiology 9, 330–348. 10.1111/j.1472-4669.2011.00281.x 21535364

[B23] Grünke S. Lichtschlag A. de Beer D. Felden J. Salman V. Ramette A. (2012). Mats of psychrophilic thiotrophic bacteria associated with cold seeps of the Barents Sea. Biogeosciences 9, 2947–2960. 10.5194/bg-9-2947-2012

[B24] Gurevich A. Saveliev V. Vyahhi N. Tesler G. (2013). QUAST: quality assessment tool for genome assemblies. Bioinformatics. 29, 1072–1075 10.1093/bioinformatics/btt086 23422339 PMC3624806

[B25] Harold R. Stanier R. Y. (1955). The genera *Leucothrix* and *Thiothrix*. Bacteriol. Rev. 19, 49–64. 13239547 10.1128/br.19.2.49-64.1955 PMC180814

[B26] Harris S. R. Feil E. J. Holden M. T. G. Quail M. A. Nickerson E. K. Chantratita N.. (2010). Evolution of MRSA during hospital transmission and intercontinental spread. Science 327, 469–474. 10.1126/science.1182395 20093474 PMC2821690

[B27] Heijs S. K. Sinninghe Damste J. S. Forney L. J. (2005). Characterization of a deep-sea microbial mat from an active cold seep at the Milano mud volcano in the Eastern Mediterranean Sea. FEMS Microbiol. Ecol. 54, 47–56. 10.1016/j.femsec.2005.02.007 16329971

[B28] Hilbert D. W. Piggot P. J. (2004). Compartmentalization of gene expression during *Bacillus subtilis* spore formaiton. Microbiol. Mol. Biol. Rev. 68, 234–262. 10.1128/MMBR.68.2.234-262.2004 15187183 PMC419919

[B29] Hinck S. Mussmann M. Salman V. Neu T. R. Lenk S. Beer D.. (2011). Vacuolated *Beggiatoa*-like filaments from different hypersaline environments form a novel genus. Environ. Microbiol. 13, 3194–3205. 10.1111/j.1462-2920.2011.02513.x 21651683

[B30] Holt K. E. Parkhill J. Mazzoni C. J. Roumagnac P. Weill F.-X. Goodhead I.. (2008). High-throughput sequencing provides insights into genome variation and evolution in *Salmonella typhi*. Nat. Genetics 40, 987–993. 10.1038/ng.195 18660809 PMC2652037

[B31] Jacq E. Prieur D. Nichols P. White D. C. Porter T. Geesey G. G. (1989). Microscopic examination and fatty-acid characterization of filamentous bacteria colonizing substrata around subtidal hydrothermal vents. Arch. Microbiol. 152, 64–71. 10.1007/BF00447013

[B32] Jannasch H. W. Nelson D. C. Wirsen C. O. (1989). Massive natural occurrence of unusually large bacteria (*Beggiatoa* sp.) at a hydrothermal deep-sea vent site. Nature 342, 834–836. 10.1038/342834a0

[B33] Jannasch H. W. Wirsen C. O. (1981). Morphological survey of microbial mats near deep-sea thermal vents. Appl. Environ. Microbiol. 41, 528–538. 16345722 10.1128/aem.41.2.528-538.1981 PMC243726

[B34] Jørgensen B. B. (1977). Distribution of colorless sulfur bacteria (*Beggiatoa* spp) in a coastal marine sediment. Mar. Biol. 41, 19–28. 10.1007/BF00390577

[B35] Jørgensen B. B. Dunker R. Grünke S. Roy H. (2010). Filamentous sulfur bacteria, *Beggiatoa* spp, in arctic marine sediments (Svalbard, 79 degrees N). FEMS Microbiol. Ecol. 73, 500–513. 10.1111/j.1574-6941.2010.00918.x 20608982

[B36] Joye S. B. Boetius A. Orcutt B. N. Montoya J. P. Schulz H. N. Erickson M. (2004). The anaerobic oxidation of methane and sulfate reduction in sediments from Gulf of Mexico cold seeps. Chem. Geol. 205, 219–238. 10.1016/j.chemgeo.2003.12.019

[B37] Joye S. B. Bowles M. W. Samarkin V. A. Hunter K. S. Niemann H. (2010). Biogeochemical signatures and microbial activity of differnet cold-seep habitats along the Gulf of Mexico deep slope. Deep Sea Res. II 57, 1990–2001. 10.1016/j.dsr2.2010.06.001

[B38] Kalanetra K. M. Huston S. L. Nelson D. C. (2004). Novel, attached, sulfur-oxidizing bacteria at shallow hydrothermal vents possess vacuoles not involved in respiratory nitrate accumulation. Appl. Environ. Microbiol. 70, 7487–7496. 10.1128/AEM.70.12.7487-7496.2004 15574952 PMC535177

[B39] Kalanetra K. M. Nelson D. C. (2010). Vacuolate-attached filaments: highly productive *Ridgeia piscesae* epibionts at the Juan de Fuca hydrothermal vents. Mar. Biol. 157, 791–800. 10.1007/s00227-009-1362-3 24391244 PMC3873080

[B40] Kearse M. Moir R. Wilson A. Stones-Havas S. Cheung M. Sturrock M.. (2012). Geneious basic: an integrated and extendable desktop software platform for the organization and analysis of sequence data. Bioinformatics 28, 1647–1649. 10.1093/bioinformatics/bts199 22543367 PMC3371832

[B41] Khakhlova O. Brock R. (2006). Elimination of deleterious mutations in plastid genomes by gene conservation. Plant J. 46, 85–94. 10.1111/j.1365-313X.2006.02673.x 16553897

[B42] Kojima H. Fukui M. (2003). Phylogenetic analysis of *Beggiatoa* spp. from organic rich sediment of Tokyo Bay, Japan. Water Res. 37, 3216–3223. 10.1016/S0043-1354(03)00206-9 14509709

[B43] Lane N. Martin W. (2010). The energetics of genome complexity. Nature 467, 929–934. 10.1038/nature09486 20962839

[B44] Larkin J. M. Henk M. C. (1996). Filamentous sulfide-oxidizing bacteria at hydrocarbon seeps of the Gulf of Mexico. Microsc. Res. Tech. 33, 23–31. 8820662 10.1002/(SICI)1097-0029(199601)33:1<23::AID-JEMT4>3.0.CO;2-1

[B45] Lasken R. S. (2007). Single-cell genomic sequencing using multiple displacement amplification. Curr. Opin. Microbiol. 10, 510–516. 10.1016/j.mib.2007.08.005 17923430

[B46] Limmathurotsakul D. Holden M. T. G. Coupland P. RPrice E. P. Chantratita N. Wuthiekanun V.. (2014). Microevolution of *Burkholderia pseudomallei* during an acute infection. J. Clin. Microbiol. 52, 3418–3421. 10.1128/JCM.01219-14 24966357 PMC4313173

[B47] Lloyd K. G. Albert D. B. Biddle J. F. Chanton J. P. Pizarro O. Teske A. (2010). Spatial structure and activity of sedimentary microbial communities underlying a *Beggiatoa* spp. mat in a Gulf of Mexico hydrocarbon seep. PLoS ONE 5:e8738. 10.1371/journal.pone.0008738 20090951 PMC2806916

[B48] Ludwig W. Strunk O. Westram R. Richter L. Meier H. Yadhukumar. (2004). ARB: a software environment for sequence data. Nucleic Acids Res. 32, 1363–1371. 10.1093/nar/gkh293 14985472 PMC390282

[B49] MacGregor B. J. Biddle J. F. Harbort C. Matthysse A. G. Teske A. (2013a). Sulfide oxidation, nitrate respiration, carbon acquisition, and electron transport pathways suggested by the draft genome of a single orange Guaymas Basin *Beggiatoa* (Cand. Maribeggiatoa) sp. filament. Mar. Genomics 11, 53–65. 10.1016/j.margen.2013.08.001 24012537

[B50] MacGregor B. J. Biddle J. F. Teske A. (2013b). Mobile elements in a single-filament orange Guaymas Basin *Beggiatoa* (“Candidatus Maribeggiatoa”) sp. draft genome: evidence for genetic exchange with cyanobacteria. Appl. Environ. Microbiol. 79, 3974–3985. 10.1128/AEM.03821-12 23603674 PMC3697557

[B51] McHatton S. C. Barry J. P. Jannasch H. W. Nelson D. C. (1996). High nitrate concentrations in vacuolate, autotrophic marine *Beggiatoa* spp. Appl. Environ. Microbiol. 62, 954–958. 16535282 10.1128/aem.62.3.954-958.1996 PMC1388807

[B52] McKay L. J. MacGregor B. J. Biddle J. F. Albert D. B. Mendlovitz H. P. Hoer D. R. (2012). Spatial heterogeneity and underlying geochemistry of phylogentically diverse orange and white *Beggiatoa* mats in Guaymas Basin hydrothermal sediments. Deep Sea Res. I 67, 21–31. 10.1016/j.dsr.2012.04.011

[B53] Mendell J. E. Clements K. D. Choat J. H. Angert E. R. (2008). Extreme polyploidy in a large bacterium. Proc. Natl. Acad. Sci. U.S.A. 105, 6730–6734. 10.1073/pnas.0707522105 18445653 PMC2373351

[B54] Mussmann M. Hu F. Z. Richter M. de Beer D. Preisler A. Jørgensen B. B.. (2007). Insights into the genome of large sulfur bacteria revealed by analysis of single filaments. PLoS Biol. 5:230. 10.1371/journal.pbio.0050230 17760503 PMC1951784

[B55] Nelson D. C. Wirsen C. O. Jannasch H. W. (1989). Characterization of large, autotrophic *Beggiatoa* spp. abundant at hydrothermal vents of the Guaymas Basin. Appl. Environ. Microbiol. 55, 2909–2917. 16348053 10.1128/aem.55.11.2909-2917.1989 PMC203190

[B56] Nelson W. C. Jannasch H. W. (1983). Chemoautotrophic growth of a marine *Beggiatoa* in sulfide-gradient cultures. Arch. Microbiol. 136, 262–269. 10.1007/BF00425214

[B57] Otto S. P. (2007). The evolutionary consequences of polyploidy. Cell 131, 452–462. 10.1016/j.cell.2007.10.022 17981114

[B58] Parks D. H. Imelfort M. Skennerton C. T. Hugenholtz P. Tyson G. W. (2015). CheckM: assessing the quality of microbial genomes recovered from isolates, single cells, and metagenomes. Peer J. Pre. Prints 25, 1043–1055. 10.1101/gr.186072.114 25977477 PMC4484387

[B59] Pinard R. de Winter A. Sarkis G. J. Gerstein M. B. Tartaro K. R. Plant R. N.. (2006). Assessment of whole genome amplification-induced bias through high-throughput, massively parallel whole genome sequencing. BMC Bioinformatics 7:216. 10.1186/1471-2164-7-216 16928277 PMC1560136

[B60] Pruesse E. Quast C. Knittel K. Fuchs B. M. Ludwig W. G. Peplies J.. (2007). SILVA: a comprehensive online resource for quality checked and aligned ribosomal RNA sequence data compatible with ARB. Nucleic Acids Res. 35, 7188–7196. 10.1093/nar/gkm864 17947321 PMC2175337

[B61] Quast C. Pruesse E. Yilmaz P. Gerken J. Schweer T. Yarza P.. (2013). The SILVA ribosomal RNA gene database project: improved data processing and web-based tools. Nucleic Acids Res. 41, D590–D596. 10.1093/nar/gks1219 23193283 PMC3531112

[B62] Raghunathan A. Ferguson H. R. Bornarth C. J. Song W. Driscoll M. Lasken R. S. (2005). Genomic DNA amplification from a single bacterium. Appl. Environ. Microbiol. 71, 3342–3347. 10.1128/AEM.71.6.3342-3347.2005 15933038 PMC1151817

[B63] Salman V. Amann R. Shub D. A. Schulz H. D. (2012). Multiple self-splicing introns in the 16S rRNA genes of giant sulfur bacteria. Proc. Natl. Acad. Sci. U.S.A. 109, 4203–4208. 10.1073/pnas.1120192109 22371583 PMC3306719

[B64] Salman V. Bailey J. V. Teske A. (2013). Phylogenetic and morphologic complexity of giant sulphur bacteria. Antonie Van Leeuwenhoek 104, 169–186. 10.1007/s10482-013-9952-y 23793621

[B65] Salman V. Yang T. Berben T. Klein F. Angert E. R. Teske A. (2015). Calcite-accumulating large sulfur bacteria of the genus *Achromatium* in Sippewissett salt marsh. ISME J. 9, 2503–2514. 10.1038/ismej.2015.62 25909974 PMC4611513

[B66] Sandh G. El-Shehawy R. Diez B. Bergman B. (2009). Temporal separation of cell division and diazotrophy in the marine diazotrophic cyanobacterium *Trichodesmium erythraeum* IMS101. FEMS Microb. Lett. 295, 281–288. 10.1111/j.1574-6968.2009.01608.x 19456868

[B67] Schulz H. N. (2006). The genus *Thiomargarita*, in The Prokaryotes, 3rd Edn., eds Dworkin M. Falkow S. Rosenberg E. Schleifer K. -H. Stackebrandt E. (New York, NY: Springer), 1156–1163. 10.1007/0-387-30746-x_47

[B68] Schulz H. N. Brinkhoff T. Ferdelman T. G. Marine M. H. Teske A. Jørgensen B. B. (1999). Dense populations of a giant sulfur bacterium in Namibian shelf sediments. Science 284, 493–495. 10.1126/science.284.5413.493 10205058

[B69] Schulz H. N. Jørgensen B. B. (2001). Big bacteria. Annu. Rev. Microbiol. 55, 105–137. 10.1146/annurev.micro.55.1.105 11544351

[B70] Schulz H. N. Schulz H. D. (2005). Large sulfur bacteria and the formation of phosphorite. Science 307, 416–418. 10.1126/science.1103096 15662012

[B71] Sheik A. R. Muller E. E. L. Audinot J.-N. Lebrun L. A. Grysan P. Guignard C.. (2016). *In situ* phenotypic heterogeneity among single cells of the filamentous bacterium *Candidatus* Microthrix parvicella. ISME J. 10, 1274–1279. 10.1038/ismej.2015.181 26505828 PMC5029219

[B72] Soppa J. (2011). Ploidy and gene conservation in Archaea. Biochem. Soc. Trans. 39, 150–154. 10.1042/BST0390150 21265763

[B73] Spits C. Le Caignec C. De Rycke M. Van Haute L. Van Steirteghem A. Liebaers I.. (2006). Whole-genome multiple displacement amplification from single cells. Nat. Protoc. 1, 1965–1970. 10.1038/nprot.2006.326 17487184

[B74] Stein J. L. (1984). Subtidal gastropods consume sulfur-oxidizing bacteria - evidence from coastal hydrothermal vents. Science 223, 696–698. 10.1126/science.223.4637.696 17841030

[B75] Strous M. Kraft B. Bisdorf R. Tegetmeyer H. E. (2012). The binning of metagenomic contigs for microbial physiology of mixed cultures. Front. Microbiol. 3:410. 10.3389/fmicb.2012.00410 23227024 PMC3514610

[B76] Teske A. Salman V. (2014). The family Beggiatoaceae, in The Prokaryotes: Gammaproteobacteria, eds Rosenberg E. Delong E. F. Lory S. Stackebrandt E. Thompson F. L. (Berlin-Heidelberg: Springer), 93–143.

[B77] Treangen T. J. Koren S. Sommer D. D. Liu B. Astrovskaya I. Ondov B.. (2013). MetAMOS: a modular and open source assembly and analysis pipeline. Genome Biol. 14:R2. 10.1186/gb-2013-14-1-r2 23320958 PMC4053804

[B78] Walsh J. B. (1995). How often do duplicated genes evolve new functions? Genetics 139, 421–428. 7705642 10.1093/genetics/139.1.421 PMC1206338

[B79] Wendel J. F. (2000). Genome evolution in polyploids. Plant Mol. Biol. 42, 225–249. 10.1023/A:1006392424384 10688139

[B80] Williams L. A. Reimers C. (1983). Role of bacterial mats in oxygen-deficient marine basins and coastal upwelling regimes: preliminary report. Geology 11, 267–269.

[B81] Williams T. M. Unz R. F. Doman J. T. (1987). Ultrastructure of *Thiothrix* spp. and “Type 021N” bacteria. Appl. Environ. Microbiol. 53, 1560–1570. 16347385 10.1128/aem.53.7.1560-1570.1987 PMC203910

[B82] Winkel M. Salman-Carvalho V. Woyke T. Richter M. Schulz-Vogt H. Flood B. (2016). Single-cell sequencing of *Thiomargarita* reveals genomic flexibility for adaptation to dynamic redox conditions. Front. Microbiol. 7:964 10.3389/fmicb.2016.00964 PMC4914600 27446006

[B83] Yilmaz S. Allgaier M. Hugenholtz P. (2010). Multiple displacement amplification compromises quantitative analysis of metagenomes. Nat. Methods 7, 943–944. 10.1038/nmeth1210-943 21116242

