# Supplementary material for: How Clonal Is Clonal? Genome Plasticity across Multicellular Segments of a “Candidatus Marithrix sp.” Filament from Sulfidic, Briny Seafloor Sediments in the Gulf of Mexico
Source: Front Microbiol. 2016 Aug 3;7:1173. doi: 10.3389/fmicb.2016.01173 (PMC4971068; doi:10.3389/fmicb.2016.01173)
Supplement: Supplementary file 1 [file DataSheet1.PDF]

Supplementary information

How clonal is clonal? Genome plasticity across multicellular segments of a "*Candidatus* Marithrix sp." filament from sulfidic, briny seafloor sediments in the Gulf of Mexico

Verena Salman-Carvalho, Eduard Fadeev, Samantha B. Joye, and Andreas Teske

Table S1 Sequence length and taxonomic diversity of "contamination" in the datasets of the individual filament segments that were removed during the binning in Metawatt. Segments 5 and 6 show a clearly increased amount and diversity of contaminating bacterial DNA compared to segments 1-4. Unbinned contigs are generally short and with low coverage across all datasets. Compared to this, all contigs that were binned into "contamination" taxa have a generally longer N50 and higher coverage. Furthermore, they affiliate to organisms of typical marine origin, so with great confidence it can be hypothesized that they were actually associated with the initial filament segment sequenced, instead of being introduced from the lab environment.

	Mb	N50	coverage	% abundance
**segment 1**				
unbinned	0.26	490	2.3	3.3
**segment 2**				
unbinned	0.33	516	2.2	3.5
**segment 3**				
unbinned	0.17	492	2	2.2
**segment 4**				
Chromatiales	0.03	2,323	116.7	0.8
unbinned	0.24	503	1.9	2.9
**segment 5**				
unclassified	0.07	10,439	588.4	2.8
Oceanospirillales	0.04	5,910	454.8	1.3
Oscillatoriophycideae	0.08	8,165	353	2.1
Myxococcales	0.03	11,362	401.5	1
Bacillales	0.11	1,994	4.4	0
Desulfobacterales	0.08	3,074	282	2.1
Sphingobacterales	0.03	5,430	372.2	1
Flavobacteriales	0.1	1,519	4.8	0.1
Chromatiales	0.03	3,232	489.5	1.1
unbinned	0.97	469	2.1	6.4
**segment 6**				
unclassified	0.04	2,959	556.9	1.7
Oceanospirillales	0.05	6,299	436.8	1.7
Methylococcales	0.06	6,292	341.9	1.5
Oscillatoriophycideae	0.08	8,539	303.9	2.2
Myxococcales	0.05	13,891	312.5	1.4

Alteromonadales	0.03	5,658	239.6	0.6
Flavobacteriales	0.03	3,423	181.1	0.4
Desulfobacterales	0.05	2,645	441.4	1.7
unbinned	0.48	481	89.1	15.9

Table S2 Average nucleotide identities calculated two-ways.

	segment 1	segment 2	segment 3	segment 4	segment 5	segment 6
**segment 1**	100					
**segment 2**	100	100				
**segment 3**	100	100	100			
**segment 4**	100	100	100	100		
**segment 5**	99.99	99.99	99.99	99.99	100	
**segment 6**	99.98	99.98	99.98	99.98	99.99	100

Table S3 The distribution of genes into subsystems as defined by the SEED viewer in RAST. Marked in grey are those categories where all six datasets contain the same amount of genes.

Subsystem	segment 1	segment 2	segment 3	segment 4	segment 5	segment 6
Cofactors, vitamins, prosthetic groups, pigments	222	218	220	241	179	152
Cell wall and Capsule	135	135	145	121	117	113
Virulence, disease, and defense	44	42	36	44	37	34
Potassium metabolism	6	6	6	6	6	6
Photosynthesis	0	0	0	0	0	0
Miscellaneous	17	17	17	16	6	13
Phages, prophages, transposable elements, plasmids	4	4	4	4	4	4
Membrane transport	65	58	57	57	44	40
Iron acquisition, and metabolism	0	0	0	0	0	0
RNA metabolism	140	138	139	144	137	92
Nucleosides and Nucleotides	58	55	58	58	38	42
Protein metabolism	244	246	249	237	223	227
Cell division and cell cycle	22	24	22	22	22	21
Motility and chemotaxis	0	0	0	0	0	0
Regulation and cell signaling	49	50	48	46	41	36
Secondary metabolism	5	5	5	5	4	0
DNA metabolism	81	81	80	81	63	52
Fatty acids, lipids, and isoprenoids	60	61	62	60	52	44
Nitrogen metabolism	42	42	42	41	16	14

Dormancy and sporulation	1	1	1	1	1	1
Respiration	80	79	79	79	71	75
Stress response	73	72	73	72	68	64
Metabolism of aromatic compounds	3	2	3	3	2	3
Amino acids and derivatives	207	205	207	207	194	185
Sulfur metabolism	33	33	33	33	30	30
Phosphorous metabolism	58	58	58	59	28	30
Carbohydrates	126	126	125	129	98	84

Table S4 Sequence-based comparison across all datasets reveals that only little sequence data is unique for each dataset. The table lists those regions that were identified as unique in each of the filament segments, and shows the annotation according to the SEED viewer - the majority of these regions represents truncated functional entities.

segment 1

Contig	Length [bp]	Function
9	44	hypothetical protein
9	161	Uncharacterized protein ImpB
13	41	hypothetical protein
18	44	hypothetical protein
22	40	hypothetical protein
22	60	hypothetical protein
43	45	hypothetical protein
47	45	hypothetical protein

segment 2

Contig	Length [bp]	Function
5	39	hypothetical protein
7	77	hypothetical protein
18	43	hypothetical protein
24	39	hypothetical protein
24	84	hypothetical protein
62	43	hypothetical protein

segment 3

Contig	Length [bp]	Function
6	51	Cell division protein FtsH (EC 3.4.24.-)
25	52	hypothetical protein
37	40	hypothetical protein
41	425	exopolysaccharide production protein ExoQ
41	46	hypothetical protein
41	86	hypothetical protein
41	277	hypothetical protein
41	68	hypothetical protein
41	452	hypothetical protein
41	195	Methionyl-tRNA formyltransferase (EC 2.1.2.9)
41	153	Methionyl-tRNA formyltransferase-like protein 2

41	233	N-Acetylneuraminate cytidylyltransferase (EC 2.7.7.43)
41	499	N-Acetylneuraminate cytidylyltransferase (EC 2.7.7.43)
41	353	N-acetylneuraminate synthase (EC 2.5.1.56)
41	348	N-acetylneuraminate synthase (EC 2.5.1.56)
41	132	PIN domain protein
41	421	polysaccharide biosynthesis protein

segment 4

Contig	Length [bp]	Function
3	41	hypothetical protein
3	142	Ribosomal-protein-alanine acetyltransferase (EC 2.3.1.128)
6	41	hypothetical protein
19	40	hypothetical protein
23	41	hypothetical protein
34	61	hypothetical protein
34	72	hypothetical protein
39	58	tRNA uridine 5-carboxymethylaminomethyl modification enzyme GidA
46	38	hypothetical protein
46	38	hypothetical protein
53	43	hypothetical protein

segment 5

Contig	Length [bp]	Function
14	45	hypothetical protein
108	38	hypothetical protein
112	364	Transcriptional repressor of the lac operon
188	41	hypothetical protein
193	39	hypothetical protein

segment 6

Contig	Length [bp]	Function
79	76	hypothetical protein
184	44	FIG015547: peptidase, M16 family
193	40	hypothetical protein
229	44	FIG015547: peptidase, M16 family

Table S5 Function-based comparison across all datasets reveals that likewise few unique functional entities could be identified for each of the datasets. This list only contains complete genes that have been checked to not contain any ortholog in the other datasets by blastp.

segment 1

Category	Subcategory	Subsystem	Role
**Clustering-based subsystems**	Cell Division	Cell Division Subsystem including YidCD	Protein YidD
**Clustering-based subsystems**	RNA metabolism	Possible RNA degradation cluster	Nitrogen regulation protein NtrC

**Membrane Transport**	Protein secretion system, Type VI	Type VI secretion systems	Uncharacterized protein ImpB
**Virulence, Disease and Defense**	Bacteriocins, ribosomally synthesized antibacterial peptides	Colicin V and Bacteriocin Production Cluster	Colicin V production protein
**Virulence, Disease and Defense**	Resistance to antibiotics and toxic compounds	Bile hydrolysis	Choloylglycine hydrolase (EC 3.5.1.24)

segment 2

Category	Subcategory	Subsystem	Role
none			

segment 3

Category	Subcategory	Subsystem	Role
**Cell Wall and Capsule**	Capsular and extracellular polysacchrides	CMP-N-acetylneuraminate Biosynthesis	N-Acetylneuraminate cytidylyltransferase (EC 2.7.7.43)
**Cell Wall and Capsule**	Capsular and extracellular polysacchrides	CMP-N-acetylneuraminate Biosynthesis	N-acetylneuraminate synthase (EC 2.5.1.56)

segment 4

Category	Subcategory	Subsystem	Role
**Carbohydrates**	Di- and oligosaccharides	Maltose and Maltodextrin Utilization	Aldose 1-epimerase (EC 5.1.3.3)
**Cofactors, Vitamins, Prosthetic Groups, Pigments**	Tetrapyrroles	Coenzyme B12 biosynthesis	Outer membrane vitamin B12 receptor BtuB

segment 5

Category	Subcategory	Subsystem	Role
**RNA Metabolism**	RNA processing and modification	tRNA modification Bacteria	FIG137478: Hypothetical protein

segment 6

Category	Subcategory	Subsystem	Role
none			

Table S6 Absolute numbers of intrasegmental sequence heterogeneity (ISH) events as revealed by mapping the read libraries of the individual segments to the assembly of segment 3 as reference. Threshold for ISH calling was a minimum coverage of 100x and variant frequency of 0.25.

segment 1	segment 2	segment 3	segment 4	segment 5	segment 6
2327	2205	2195	1824	2050	2967
